# The relationship between stochastic and deterministic quasi-steady state approximations

**DOI:** 10.1186/s12918-015-0218-3

**Published:** 2015-11-23

**Authors:** Jae Kyoung Kim, Krešimir Josić, Matthew R. Bennett

**Affiliations:** Department of Mathematical Sciences, Korea Advanced Institute of Science and Technology, 291 Daehak-ro Yuseong-gu, Daejeon, 305-701 Korea; Mathematical Biosciences Institute, The Ohio State University, 1735 Neil Avenue, OH 43210 Columbus, USA; Department of Mathematics, University of Houston, 4800 Calhoun Rd, Houston, TX 77204-3008 USA; Department of Biology and Biochemistry, University of Houston, 4800 Calhoun Rd, Houston, TX 77204–3008 USA; Department of Biosciences, Rice University, 6100 Main St, Houston, 77005–1892 TX USA; Department of Bioengineering, Rice University, 6100 Main St, Houston, TX 77005–1892 USA

**Keywords:** Stochastic QSSA, Multi-scale stochastic simulation, Hill function, Michaelis-Menten function

## Abstract

**Background:**

The quasi steady-state approximation (QSSA) is frequently used to reduce deterministic models of biochemical networks. The resulting equations provide a simplified description of the network in terms of non-elementary reaction functions (e.g. Hill functions). Such deterministic reductions are frequently a basis for heuristic stochastic models in which non-elementary reaction functions are used to define reaction propensities. Despite their popularity, it remains unclear when such stochastic reductions are valid. It is frequently assumed that the stochastic reduction can be trusted whenever its deterministic counterpart is accurate. However, a number of recent examples show that this is not necessarily the case.

**Results:**

Here we explain the origin of these discrepancies, and demonstrate a clear relationship between the accuracy of the deterministic and the stochastic QSSA for examples widely used in biological systems. With an analysis of a two-state promoter model, and numerical simulations for a variety of other models, we find that the stochastic QSSA is accurate whenever its deterministic counterpart provides an accurate approximation over a range of initial conditions which cover the likely fluctuations from the quasi steady-state (QSS). We conjecture that this relationship provides a simple and computationally inexpensive way to test the accuracy of reduced stochastic models using deterministic simulations.

**Conclusions:**

The stochastic QSSA is one of the most popular multi-scale stochastic simulation methods. While the use of QSSA, and the resulting non-elementary functions has been justified in the deterministic case, it is not clear when their stochastic counterparts are accurate. In this study, we show how the accuracy of the stochastic QSSA can be tested using their deterministic counterparts providing a concrete method to test when non-elementary rate functions can be used in stochastic simulations.

**Electronic supplementary material:**

The online version of this article (doi:10.1186/s12918-015-0218-3) contains supplementary material, which is available to authorized users.

## Background

Biochemical systems frequently consist of reactions evolving on disparate timescales. The species regulated by fast reactions quickly equilibrate to a “quasi-steady-state (QSS)” [1], and hence these fast species can be assumed to be in a quasi-equilibrium that is dependent on the state of the slow species. This assumption allows one to eliminate the variables describing the fast species from deterministic models via non-elementary reaction functions. The deterministic quasi-state-state approximation (QSSA) can thus be used to reduce the dimensionality of a system and avoid stiffness in numerical simulations. QSSA has been widely used in both numerical and theoretical studies and its validity condition in deterministic models is well understood [1–11].

Timescale separation has also been used to reduce and accelerate simulations of stochastic models [12–30]. The QSS of a fast species in the chemical master equation (CME) can be defined as the conditional average of the species which depends on the instantaneous state of the slow species [16–18]. This approximation obviates the need to simulate fast reactions explicitly. However, to calculate the averages of the fast species, knowledge of the solution to the full CME is generally required, giving rise to a “chicken or the egg” problem – one introduces a reduced system to avoid solving the full system, but carrying out the reduction requires solving the full system. As an alternative, it has been proposed that one can approximate the needed averages using the QSS of the fast species obtained from the corresponding deterministic systems [16, 23, 24, 26]. For instance, Michaelis-Menten or Hill functions derived using the deterministic QSSA have been used as propensity functions in stochastic simulations – a method known as “stochastic QSSA”.

The validity of the stochastic QSSA relies on two assumptions: 1) the separation of timescales between the slow and fast reactions, and 2) the accurate approximation of the stochastic QSS (i.e. the conditional average of the fast species) by the deterministic QSS [30]. It is not well understood when these assumptions hold. In many previous studies, it has been assumed that the stochastic QSSA is accurate whenever the corresponding deterministic QSSA is accurate [31–34]. However, recently introduced examples show that this may not always be true, as the reduced stochastic model may poorly approximate the full model even when their deterministic counterparts agree [27, 30, 35, 36]. Due to this discrepancy, previous studies concluded that the stochastic QSSA cannot be validated using the deterministic QSSA, leaving open the question of alternative validation methods. Nevertheless, the stochastic QSSA is still used widely in simulations of complex models that would otherwise be intractable [31–34, 37–47].

Here, we identify a clear correspondence between the validities of the deterministic and the stochastic QSSA for examples widely used in biological systems. This relation provides a simple and computationally inexpensive method for validating the stochastic QSSA. Specifically, we find that discrepancies between the stochastic and the deterministic QSSA stem from the fact that, due to the random fluctuations, the stochastic system explores a wider range of states than its deterministic counterpart. We provide an analysis of a two-state promoter model, and use numerical simulations for a variety of other models to show that the stochastic QSSA is accurate only when the deterministic QSSA is accurate over a range of initial conditions that cover the most likely states explored by the stochastic system. Our finding suggests that in many cases the validity of the stochastic QSSA can be checked post facto by examining the deterministic QSSA over a range of initial conditions obtained by simulating the reduced stochastic model.

## Results and discussion

### Discrepancy between stochastic and deterministic QSSA

**Fig. 1:**
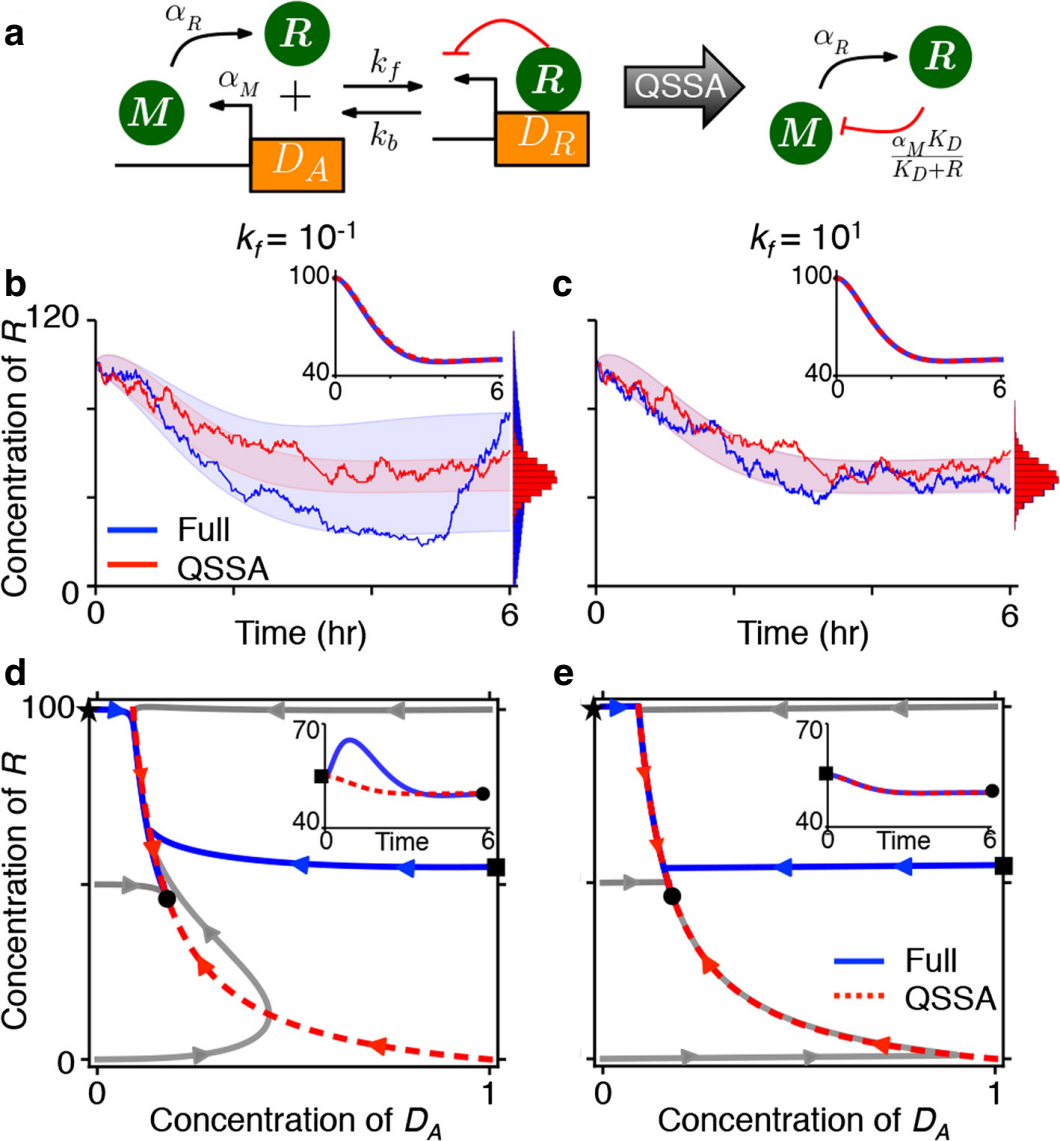
The relationship between the accuracy of the deterministic and the stochastic QSSA. **a** The diagrams for the full model (Eqs. 1–3) and the reduced model (Eq. 5). **b**–**c** The deterministic QSSA is accurate when both *k*_*f*_=10^−1^ *h* ^−1^ and *k*_*f*_=10^1^ *h* ^−1^ (the insets). However, the corresponding stochastic QSSA is accurate only when *k*_*f*_=10^1^. The colored ranges and histograms represent a standard deviation of *R* from its mean and the distribution of *R* at steady state, respectively. Here, *K*_*D*_=10, *α*_*M*_=300*h* ^−1^, *β*_*M*_=*β*_*R*_=1*h* ^−1^. *M*(0)=*R*(0)=100 and *D*_*A*_(0)=0. **d**–**e** The phase plots of deterministic trajectories with various initial conditions when *k*_*f*_=10^−1^ (**d**) and *k*_*f*_=10^1^ (**e**). The star and the square indicate initial conditions used in (**b**) and (**c**) and the insets of (**d**) and (**e**), respectively. The black circle indicates the fixed point. Here, we assumed *M*(0)=*R*(0)

We began investigating the relationship between the stochastic and the deterministic QSSA with a simple transcriptional negative feedback loop model (Fig. 1a):

(1) $$\begin{array}{*{20}l} \dot {M} &= \alpha_{M} D_{A} - \beta_{M} M, \end{array} $$

(2) $$\begin{array}{*{20}l} \dot {R} &= \alpha_{R} M - \beta_{R} R -k_{f} R D_{A} + k_{b} D_{R}, \end{array} $$

(3) $$\begin{array}{*{20}l} \dot {D}_{A} &= -k_{f} R D_{A} + k_{b} D_{R}, \end{array} $$

where *M*, *R*, *D*_*A*_, and *D*_*R*_ are the concentrations of mRNA, repressor, active DNA, and repressed DNA, respectively, measured relative to the total amount of DNA, *D*_*T*_; *α*_*M*_ and *α*_*R*_ are the transcription and translation rates, respectively; *k*_*f*_ and *k*_*r*_ are the forward and reverse rates of repressor binding to DNA; and *β*_*M*_ and *β*_*R*_ are the degradation rates of mRNA and repressor, respectively. The total amount of DNA is conserved, and hence our choice of units implies that *D*_*A*_+*D*_*R*_=1.

If *D*_*A*_ evolves faster than *M* and *R*, Eq. 3 equilibrates faster than Eqs. 1-2. Thus, *D*_*A*_ can be assumed to be in steady state with respect to the instantaneous state of *M* and *R* [1, 4–8, 10]. The QSS of *D*_*A*_ can be obtained by solving the QSS equation, $\dot {D}_{A}=0$, giving

(4) $$ D_{A} (R) = \frac {K_{D}}{K_{D}+R},\  $$

where *K*_*D*_=*k*_*b*_/*k*_*f*_. Equation 4 allows us to close the remaining equation (Eqs. 1–2) giving the reduced system (Fig. 1a):

(5) $$\begin{array}{*{20}l} \dot {M} &= \alpha_{M} \frac {K_{D}}{R + K_{D}} - \beta_{M} M,\\ \dot {R} &= \alpha_{R} M - \beta_{R} R.\ \end{array} $$

Note that different choices of *k*_*b*_ and *k*_*f*_ for Eqs. 1–3 result in the same reduction (Eq. 5), provided the ratio *K*_*D*_=*k*_*b*_/*k*_*f*_ is fixed. We asked whether values of *k*_*f*_ that lead to accurate deterministic reductions also provide accurate stochastic approximations. To address this question we varied *k*_*f*_ while keeping *K*_*D*_=10 fixed. For both *k*_*f*_=10^−1^ and *k*_*f*_=10^1^, the reduced model provides an accurate deterministic approximation to the full model for certain initial condition (insets of Fig. 1b and c). However, the corresponding stochastic simulations of the reduced and the full model disagree when *k*_*f*_=10^−1^ (Fig. 1b and c) (see ‘Methods’ for details of stochastic simulations). These results agree with previous studies showing that the accuracy of the deterministic reduced model does not guarantee the accuracy of stochastic simulations [27, 30, 35, 36].

Interestingly, the reduced deterministic model becomes inaccurate when initial conditions are changed for *k*_*f*_=10^−1^, but remains accurate over a wide range of initial conditions when *k*_*f*_=10^1^ (Fig. 1d and e). Thus, we hypothesized that the stochastic reduction is accurate if the deterministic reduction is valid over a range of initial conditions. We next investigated this hypothesis analytically and numerically.

### A condition for an accurate stochastic QSSA

In the presence of timescale separation, deterministic solutions evolve in two phases: an initial transient (IT) phase and a QSS phase. The two phases correspond to times before and after the solutions are in QSS, respectively. During the IT phase the solution approaches the slow manifold defined by the QSS equation (e.g. the red dashed lines in Fig. 1d and e). In this phase of the full model the “fast” variables have not equilibrated to their QSS. In the reduced model, however, fast variables are assumed to equilibrate instantaneously. Thus, the reduction is valid only when the “slow” variables change little during the IT phase [1, 4]. If this condition is satisfied, trajectories in the phase plane horizontally approach the slow manifold from their initial conditions (Fig. 1d and e) [1, 3, 4].

The trajectories of the deterministic system evolve along the slow manifold during the QSS phase (Fig. 1d and e). However, trajectories of the corresponding stochastic system fluctuate around the slow manifold. Therefore, any trajectory starting away from the slow manifold that does not horizontally relax onto it on average will contribute to the error in the stochastic QSSA. Thus, during the return of the fast variables to the slow manifold after a random fluctuation, the slow variables should change little. This leads to the hypothesis that for the *stochastic* QSSA to be accurate, the *deterministic* QSSA should be accurate for a range of initial conditions which contain the most likely fluctuations away from the slow manifold.

To investigate this hypothesis, following Segel and Slemrod [1], we estimated how much a slow variable (*R*) changes compared to its initial condition (*R*(0)) during the IT phase:

(6) $$ \left|\frac{\Delta R}{R(0)}\right| \approx \frac{\beta_{R} / k_{f} +D_{A}^{\text{max}}}{R(0)+K_{D}}.  \  $$

Here $D_{A}^{max}$ is the maximum value of *D*_*A*_ during the IT phase (See ‘Methods’ for details). This quantity should be small for the deterministic QSSA to be accurate. Equation 6 predicts that the error of the deterministic QSSA increases as either *R*(0) or *k*_*f*_ decrease, in agreement with simulations (Fig. 1d and e).

In previous work [30] we showed that the stochastic QSSA is accurate when (See ‘Methods’ for details):

(7) $$ k_{f} \gg \beta_{R} \qquad \text{and} \qquad \frac{\text{Var}(D_{A})}{R+K_{D}} \ll1. \  $$

The first condition guarantees that the reactions regulating *D*_*A*_ is fast and the second condition ensures that the stochastic QSS of *D*_*A*_ (i.e. the conditional average of *D*_*A*_) is accurately approximated by a deterministic QSS equation for *D*_*A*_ (Eq. 4). Interestingly, the second condition is similar in form to the condition for timescale separation for the deterministic QSSA during the IT phases (Eq. 6). Thus, the conditions that imply the accuracy of the stochastic QSSA (Eq. 7) are satisfied if Eq. 6 is small for those values of *R*(0) that cover the range of states realized during the stochastic simulations. This supports our hypothesis that the stochastic QSSA is accurate when the deterministic QSSA is accurate for a range of initial conditions. This also explains why the parameter region for which the stochastic QSSA is valid is smaller than for the deterministic QSSA (Fig. 1b and c).

### A numerical method for testing the accuracy of the stochastic QSSA

Equations 6 and 7 predict that both the stochastic and the deterministic QSSA become increasingly accurate as *k*_*f*_ or *K*_*D*_ increases. We tested these predictions by numerically estimating the error of the deterministic QSSA using:

(8) $$ \max_{\substack{\mathcal{N}}}\frac {{\int_{0}^{T}} \! \left|X_{\text{full}} (t) - X_{\text{QSSA}} (t)\right| dt}{{\int_{0}^{T}} \! \left|X_{\text{QSSA}} (t)\right| dt}, \  $$

where *X*_full_(*t*) and *X*_QSSA_(*t*) represent the solutions (*R*(*t*)) of the full (Eqs. 1–3) and the reduced model (Eq. 5), respectively. We chose the range of initial conditions ($\mathcal {N}$) to be three standard deviations from the expected values of the slow variables (*M* and *R*) at steady state of the reduced model (Red histograms in Fig. 1b and c). The entire range of the fast variable (*D*_*A*_) was included in $\mathcal {N}$ because we cannot determine its expectation and standard deviation with the reduced model. Equation 8 provides a measure of the accuracy of the deterministic QSSA over the range of initial conditions that contains the likely stochastic perturbations from the steady state.

**Fig. 2:**
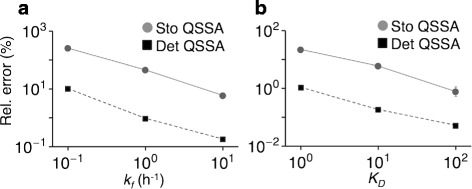
The numerically estimated error of deterministic and stochastic QSSA show strong correlation. **a**–**b** The error of the stochastic QSSA is closely corelated to the error in the deterministic QSSA when *k*_*f*_ (**a**) and *K*_*D*_ (**b**) change. Here, the error in the deterministic QSSA is measured using the relative differences in deterministic solutions *R*(*t*) given in Eq. 8. The error of the stochastic QSSA is measured using the relative difference of coefficient of variation at steady state between the full and the reduced model. Note that since the error of stochastic and deterministic QSSA were measured in different ways, they can potentially differ, even by orders of magnitude. See Additional file 1: Figure S2 for the difference of the distributions of *R* between the stochastic QSSA and full model. The error bars were estimated with the boot strap method as described in ‘Methods’. See Fig. 1 for the details of parameters

We used the relative error of the coefficient of variation at steady state to test the accuracy of the stochastic QSSA. Both Eq. 8 and the error of the stochastic QSSA decrease as *k*_*f*_ and *K*_*D*_ increase (Fig. 2), matching our analysis (Eqs. 6–7). This again shows the close relation between the error of deterministic and the stochastic QSSA. In more complex models an analytical approach may not be possible. For such models, the accuracy of the deterministic QSSA estimated numerically using Eq. 8 can inform the validity of the stochastic QSSA as described in the next section.

The proposed numerical method for testing the accuracy of the stochastic QSSA with Eq. (8) is simple, but has limits. Importantly, we do not require stochastic simulations of the full model which can be computationally expensive. Hence, the distribution and the range of fluctuations of the fast species is not required to be known. Our method requires that the reduced deterministic system is accurately approximated the full deterministic system over a range of initial conditions that includes all plausible values of the fast species. This range of initial conditions must include most of the mass of the distribution of the fast species, but can be larger. Since we do not know the distribution of the fast species this could include initial conditions that are outside the fluctuation range of the fast species. Hence, our condition can be too restrictive, as we could require it to hold for initial conditions that are never visited by the stochastic system.

As an example, consider the unscaled model in Eqs. 1–3 (i.e. variables are not scaled by *D*_*T*_). For fixed volume *Ω*, as *D*_*T*_ increases the number of molecules of the fast species, *D*_*A*_, increases. This reduces fluctuations of *D*_*A*_ and hence the range of the likely values of *D*_*A*_ (Additional file 1: Figure S1A). Thus, as *D*_*T*_ increases, requiring the deterministic QSSA to be accurate for all possible values of *D*_*A*_ is a more restrictive condition than necessary to ensure the accuracy of the stochastic QSSA. As a result, when the error of the deterministic QSSA is estimated for a range of initial conditions that includes all possible values of the fast species, the error of the deterministic and stochastic QSSA shows a discrepancy as *D*_*T*_ increases (Additional file 1: Figure S1B), and our method will give a false negative. This obvious limitation of our method can be resolved by estimating the distribution of *D*_*A*_ at least approximately.

We also found that normalizing the fast species as we scaled the system with *D*_*T*_ can help overcome the limitation of our method. In the scaled system (Eqs. 1–3 and Eq. 5), *D*_*T*_ only appears implicitly in the parameter $k_{f}=D_{T} k_{f}^{*}$ ($k_{f}^{*}$ is the binding rate before scale) instead of directly affecting the DNA concentration. In the scaled system, our method predicts that as *k*_*f*_ increases, the stochastic QSSA becomes more accurate (Fig. 2a). This result can be used to predict that, since $k_{f}=D_{T} k_{f}^{*}$ as either $k_{f}^{*}$ or *D*_*T*_ increases, the accuracy of the stochastic QSSA for the unscaled model would equally increase. This agrees with the numerical error analysis of the stochastic QSSA for the original model (Additional file 1: Figure S1C). In summary, the effect of *D*_*T*_ on the accuracy of the unscaled model can be accurately captured by applying our method Eq. 8 to the scaled model. Hence, when testing the parameter dependence of the stochastic QSSA, normalization of the fast species can improve the reliability of our criterion using Eq. 8.

### Key parameters for an accurate stochastic QSSA

**Fig. 3:**
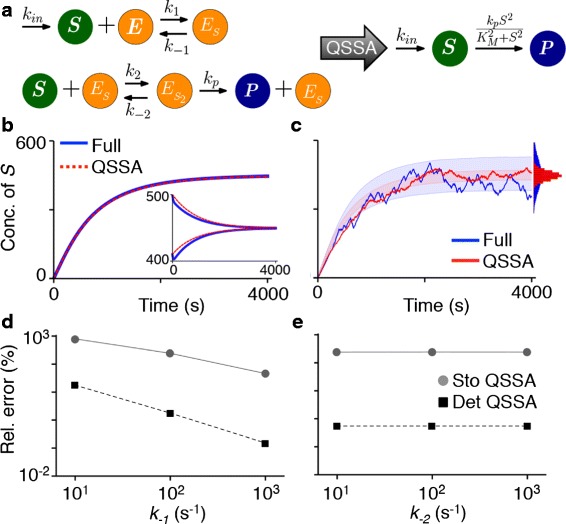
The deterministic QSSA can identify key parameters determining the accuracy of the stochastic QSSA. **a** The diagrams of the full model (Eqs. 9) and the reduced model (Eq. 10). **b**–**c)** Whereas the deterministic QSSA is accurate (**b**), but the stochastic QSSA is inaccurate (**c**) with the same initial condition: $ S(0)=E_{S}(0)=E_{S_{2}}(0)=0\protect \phantom {\dot {i}\!}$. The deterministic QSSA becomes inaccurate with a different initial condition: *S*(0)=410 or 500, *E*_*S*_(0)=0, $E_{S_{2}}(0)=1$ (the inset of (**b**)). The colored ranges and histograms represent a standard deviation of *S* from its mean and the distribution of *S* at steady state, respectively (**c**). The parameters of the model is adopted from Thomas et al. [27]: *k*_*in*_=0.5*s* ^−1^, *k*_−1_=*k*_−2_=100*s* ^−1^, *k*_*p*_=1*s* ^−1^, $K_{m_{1}}=2 \cdot 10^{6}$, $K_{m_{2}}=0.101$. **d**–**e** Both the errors of the stochastic and the deterministic QSSA depends on *k*_−1_ (**d**), but not *k*_−2_ (**e**). The errors were measured as in Fig. 2. In particular, the error of the deterministic QSSA is estimated with Eq. 8, where *T*=4000 and *X*(*t*)=*S*(*t*) were used. See Additional file 1: Figure S3 for the distribution of *S* from the stochastic simulations

Next, we tested whether our finding can explain the previously observed discrepancies between the stochastic and the deterministic QSSA [27, 30]. We began with a model of cooperative enzyme kinetics (Fig. 3a) [5, 27]:

(9) $$ \begin{aligned} \dot {S} &= k_{in} - k_{1} SE + k_{-1}E_{S} - k_{2}SE_{S} +k_{-2}E_{S_{2}}, \\ \dot {E_{S}} &= k_{1} SE - k_{-1}E_{S} - k_{2}SE_{S} +k_{-2}E_{S_{2}}+k_{p} E_{S_{2}}, \\ \dot {E}_{S_{2}} &= k_{2}SE_{S} - k_{-2}E_{S_{2}} - k_{p} E_{S_{2}},\\ \dot {P} &= k_{p} E_{S_{2}}.\ \end{aligned}  $$

Here, the substrate (*S*) reversibly binds with enzyme (*E*) to form a complex (*E*_*S*_). This complex can bind another substrate (*S*) to form a second complex ($E_{S_{2}}$), which dissociates to the first complex (*E*_*S*_) and product (*P*). We scale all concentration relative to the total enzyme concentration, *E*_*T*_. The total enzyme concentration is constant, so that $E+E_{S}+E_{S_{2}}=1\phantom {\dot {i}\!}$.

If *E*_*S*_ and $E_{S_{2}}$ equilibrate more quickly than *S*, and $\frac {k_{-1}}{k_{1}} \gg \frac {k_{-2}+k_{p}}{k_{2}}$ leading cooperative substrate binding, then the full model can be reduced with QSSA [5, 8, 27] giving:

(10) $$ \dot {S} = k_{in} - \frac{k_{p} S^{2}}{{K^{2}_{m}} + S^{2}}, \qquad \quad \dot {P} = \frac{k_{p} S^{2}}{{K^{2}_{m}} + S^{2}},  $$

where ${K^{2}_{m}} =\frac {k_{-1}}{k_{1}} \frac {k_{-2}+k_{p}}{k_{2}}$ (Fig. 3a). Thomas et al. [27] showed that, in the deterministic case, the reduced model accurately captures the behavior of the full model (Fig. 3b), but its stochastic equivalent with the same initial condition does not (Fig. 3c). This discrepancy between the deterministic and the stochastic QSSA can be explained by observing that the deterministic QSSA becomes inaccurate for other initial conditions that correspond to fluctuations from the slow manifold in the stochastic system (see the inset of Fig. 3b).

If we change *k*_−1_ and *k*_−2_ while fixing the values of $K_{m_{1}}=\frac {k_{-1}}{k_{1}}$ and $K_{m_{2}}=\frac {k_{-2}+k_{p}}{k_{2}} $, then ${K^{2}_{m}}$ in Eq. 10 does not change and the full model has the same reduction. We varied *k*_−1_ and *k*_−2_ while keeping ${K_{m}^{2}}$ fixed to determine when the reduced model is accurate. The error of the deterministic QSSA estimated with Eq. 8 decreases with the increase of *k*_−1_, but not *k*_−2_ (Fig. 3d and e). This suggests that the error of the stochastic reduction will depend on *k*_−1_, but not on *k*_−2_. We confirmed this prediction in further stochastic simulations (Fig. 3d and e). This illustrates how the key parameters determining the accuracy of the stochastic QSSA can be identified by examining the accuracy of the deterministic QSSA over an appropriate range of initial conditions.

### Comparison of different stochastic QSSAs

**Fig. 4:**
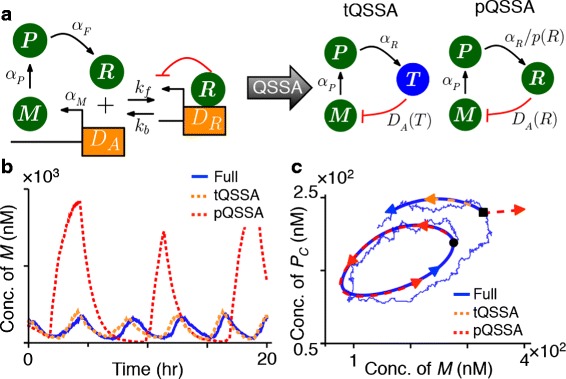
Different types of stochastic QSSA. **a** The diagram of full model (Eqs. 11-14) and two types of reduced models (Eqs. 17 and 18). **b** When an initial condition is on the limit cycle, only the stochastic tQSSA is accurate while both deterministic pQSSA and tQSSA are accurate (Additional file 1: Figure S4A). A detailed numerical error analysis with Fourier transform is provided in Additional file 1: Figure S5. **c** Even with an initial condition on the limit cycle (black circle), for which deterministic pQSSA is accurate (Additional file 1: Figure S4A), the stochastic trajectory escapes the limit cycle due to the fluctuation, where the deterministic pQSSA is inaccurate (e.g. black square) (Additional file 1: Figure S4B). The parameters of the model are adopted from [30]: *α*_*M*_=15.1745*h* ^−1^, *α*_*P*_=*α*_*F*_=*β*_*M*_=*β*_*P*_=*β*_*F*_=1*h* ^−1^, *k*_*b*_=50*h* ^−1^, *k*_*f*_=200*n* *M* ^−1^ *h* ^−1^, *D*_*T*_=164.75*n* *M*

Up to this point we have only considered the standard QSSA (sQSSA), but other versions of QSSAs have been proposed [4, 6, 7, 10, 12, 30]. Therefore, we next investigated whether the same relationship between the stochastic and the deterministic QSSA holds for other QSS reduction techniques, such as the total QSSA (tQSSA) [4, 6, 10] and the pre-factor QSSA (pQSSA) [7, 10, 12]. For illustration, we chose a transcriptional negative feedback loop model (Fig. 4a) given by the system:

(11) $$\begin{array}{*{20}l} \dot {M} &=\, \alpha_{M} D_{A} - \beta_{M} M, \end{array} $$

(12) $$\begin{array}{*{20}l} \dot {P} &=\, \alpha_{P} M - \beta_{P} P, \end{array} $$

(13) $$\begin{array}{*{20}l} \dot {R} &=\, \alpha_{R} P - \beta_{R} R -k_{f} R D_{A} + k_{b} D_{R}, \end{array} $$

(14) $$\begin{array}{*{20}l} \dot {D}_{R} &= k_{f} R D_{A} - k_{b} D_{R} - \beta_{R} D_{R},\ \end{array} $$

where the transcription of mRNA (*M*) occurs proportional to active DNA (*D*_*A*_) and *M* is translated to the protein (*P*), which transforms to the active repressor (*R*). The repressor reversibly binds with *D*_*A*_ to form repressed DNA (*D*_*R*_). The total DNA concentration *D*_*T*_=*D*_*A*_+*D*_*R*_ is constant.

Our previous study shows that the tQSSA, but not the sQSSA, provides a valid reduction of the full model when binding and unbinding are fast [30]. For the tQSSA, we introduce a new variable, the total amount of repressor, *T*≡*R*+*D*_*R*_, and replace Eqs. 13 and 14 with

(15) $$ \begin{aligned} \dot T &= \alpha_{R} P - \beta_{R} T, \\ \dot D_{R} &= k_{f} (T-D_{R}) D_{A} - k_{b} D_{R} - \beta_{R} D_{R}.\ \end{aligned}  $$

Note that *T* does not depend on the fast reversible binding unlike *F*. By using *D*_*R*_=*D*_*T*_−*D*_*A*_ and solving the QSS equations for the fast species ($\dot {D}_{R}=0$), we can obtain the equilibrium values of *D*_*A*_ in terms of *T* [30, 48]:

(16) $$ D_{A} (T) = \frac{D_{T} - T - K_{d}+ \sqrt{\left(D_{T} - T - K_{d}\right)^{2} + 4D_{T} K_{d}}}{2},   $$

where *K*_*d*_=(*k*_*f*_+*β*_*R*_)/*k*_*b*_. With this equation, we can reduce the system (Fig. 4a):

(17) $$ \begin{aligned} \dot M =&\, \alpha_{M} D_{A} (T) - \beta_{M} M,\\ \dot P =&\, \alpha_{P} M - \beta_{P} P,\\ \dot T =&\, \alpha_{R} P - \beta_{R} T.\ \end{aligned}  $$

Because the tQSS solution (Eq. 16) is less intuitive than the Michaelis-Menten-like form of the sQSS solution (Eq. 4), the reduced system can be transformed into a more intuitive form by expressing Eq. 17 in terms of the original free protein variable, *R*. Using $D_{A}(R)= \frac {D_{T} K_{d}}{R + K_{d}} $ and $\dot {T}=\frac {\partial T}{\partial R} \dot {R}$, we obtain:

(18) $$ \begin{aligned} \dot {M} =&\, \alpha_{M} \frac {D_{T} K_{d}}{R + K_{d}} - \beta_{M} M,\\ \dot {P} =&\, \alpha_{P} M - \beta_{P} P,\\ p(R) \dot {R} =&\, \alpha_{R} P - \beta_{R} \left(R + \frac {D_{T} R}{R + K_{d}}\right),\ \end{aligned}  $$

where $p(R) \equiv \frac {\partial T}{\partial R} = \frac {\partial R}{\partial R}+\frac {\partial D_{R}}{\partial R}=1+ \frac {D_{T} K_{d}}{(R + K_{d})^{2}}$ (Fig. 4a). Due to the pre-factor (*p*(*R*)) in Eq. 18, this reduction is known as pQSSA [7, 10, 12]. We have shown previously [30] that both the tQSSA and the pQSSA accurately approximate sustained oscillations of the full deterministic model (Additional file 1: Figure S4A). However, only the tQSSA provides an accurate approximation to the full stochastic model (Fig. 4b and Additional file 1: Figure S5).

The discrepancy between the stochastic and the deterministic pQSSA can again be explained by examining the initial transient for a range of initial conditions (Fig. 4c). In the deterministic case, initial conditions on the limit cycle (e.g. the black circle on blue loop in Fig. 4c) lead to solutions that are accurately approximated using the pQSSA (Additional file 1: Figure S4A). However, initial conditions off the limit cycle (e.g. the black square in Fig. 4c), the deterministic pQSSA becomes inaccurate (Additional file 1: Figure S4B). We thus expect the stochastic pQSSA to fail as well even for initial conditions on limit cycle (Fig. 4b). For a stochastic reduction to be accurate, the corresponding reduced deterministic model needs to agree with the full model in a neighborhood of the limit cycle – a neighborhood that contains most of the mass of the stationary distribution of the stochastic system. This indicates that accurately approximating limit cycle period or amplitude – the focus of most previous deterministic models [5, 7, 8, 48, 49] – is not sufficient to guarantee an accurate stochastic approximation [50]. It is also necessary to check whether the deterministic model captures global dynamical features around the neighborhood of limit cycle [51–54].

Both the deterministic pQSSA and tQSSA have been developed to improve the accuracy of sQSSA [4, 6, 7, 10]. The deterministic tQSSA broadens the range of initial conditions over which the approximation is valid during both the IT phase and QSS phase [4, 6, 10]. On the other hand, the transformations required for obtaining the pQSSA assume that the fast variables are initially in QSS (note $D_{A}(R)= \frac {D_{T} K_{d}}{R + K_{d}} $ was used) [7, 10]. Therefore, the pQSSA is not necessarily valid during the IT period unlike tQSSA. This explains why the stochastic tQSSA, but not the stochastic pQSSA, is more accurate than the stochastic sQSSA [23, 24, 30].

### Stochastic QSSA with composite reductions

**Fig. 5:**
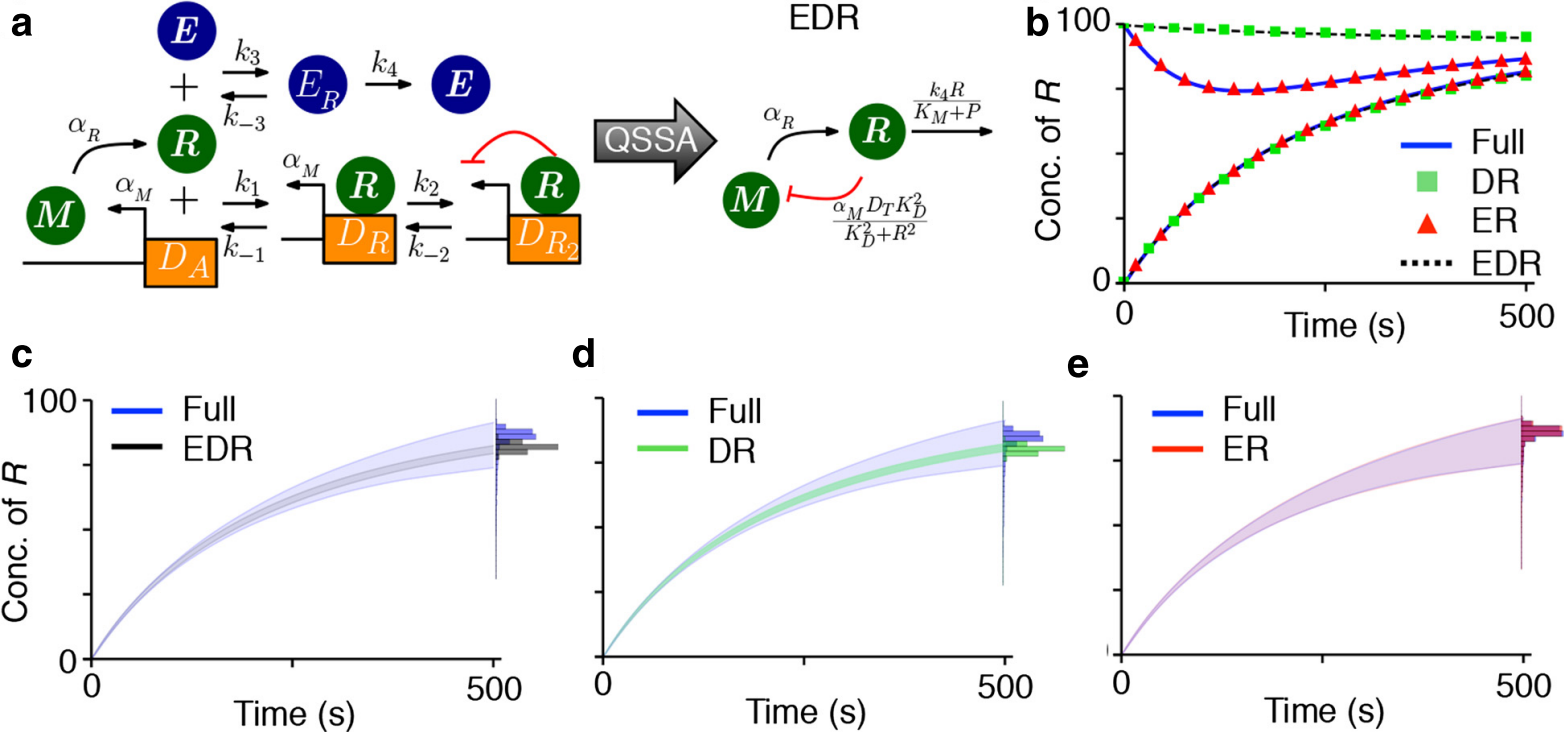
The deterministic QSSA can inform the validity of the stochastic QSSA with composite reduction. **a** The diagram of the full model (Eqs. 19) and the reduced model (Eqs. 20). **b** The three different types of reduced models (ER, DR and EDR) (Additional file 1: Figure S6) accurately approximate the deterministic simulations of full model with an initial condition, which will be used for stochastic simulation: *M*(0)=*R*(0)=*E*_*R*_(0)=*G*(0)=*G*_*R*_(0)=0. However, only ER model accurately approximates the full model if *R*(0)=100. **c**–**e** The stochastic simulation of only ER model is accurate for an initial condition with which all three types of reduced model are deterministically accurate (Fig. 5b). The colored ranges and histograms represent a standard deviation of *R* from its mean and the distribution of *R* at the steady state. The parameters of the model are adopted from [27]: *α*_*M*_=50*s* ^−1^, *β*_*M*_=*k*_*R*_=*k*_4_=1*s* ^−1^, *k*_−1_=*k*_−2_=*k*_−3_=10*s* ^−1^, *k*_1_=10^−5^ *s* ^−1^, *k*_2_=10^2^ *s* ^−1^, *K*_*M*_=110, *D*_*T*_=0.01

We next investigated whether our findings can be extended to more complex models that contain multiple non-elementary functions. We considered the transcriptional negative feedback loop model with enzymatic degradation (Fig. 5a) can be described using the following equations [27]:

(19) $$ \begin{aligned} \dot {M} &= \alpha_{M}(D+D_{R}) -\beta_{M} M,\\ \dot {R} &= \alpha_{R} M -k_{1} RD + k_{-1}D_{R} -k_{2} R D_{R} \\ &\quad+k_{-2}D_{R_{2}} - k_{3}ER + k_{-3}E_{R}, \\ \dot {D} &= -k_{1} RD + k_{-1}D_{R}, \\ \dot {D_{R}} &= k_{1} RD - k_{-1}D_{R} -k_{2} RD_{R} + k_{-2}D_{R_{2}}, \\ \dot {E_{R}} &= k_{3}ER - k_{-3}E_{R} -k_{4}E_{R}. \end{aligned}  $$

The rate of mRNA (*M*) transcription is proportional to the concentration of unbound DNA (*D*), and DNA bound with one protein (*D*_*R*_). DNA bound with two proteins ($D_{R_{2}}$) is reposed status. mRNA is translated to protein (*R*), which reversibly binds with *D* and *D*_*R*_ to form *D*_*R*_ and $D_{R_{2}}$, respectively. The protein also reversibly binds with the enzyme (*E*) to form a complex (*E*_*R*_), which decays. $D_{T} = D+D_{R}+D_{R_{2}}\phantom {\dot {i}\!}$ and *E*_*T*_=*E*+*E*_*R*_ are constant. If *E*_*R*_, *D*, and *D*_*R*_ equilibrate faster than *M* and *R*, and $\frac {k_{-1}}{k_{1}} \gg \frac {k_{-2}}{k_{2}}$, the model can be reduced to [27]):

(20) $$ \dot {M} = \frac{\alpha_{M} D_{T} {K^{2}_{D}}}{{K^{2}_{D}} +R^{2}} - \beta_{M}M, \quad \dot {R} = k_{R} M - \frac{k_{4} E_{T} R}{K_{M} + R},  $$

where *K*_*M*_=(*k*_−3_+*k*_4_)/*k*_3_ and ${K^{2}_{D}} =k_{-1}k_{-2}/k_{1}k_{2}$ [27]. The reduced model is obtained through composite reductions resulting in two non-elementary terms: the Hill function, $ \frac {\alpha _{M} D_{T} {K^{2}_{G}}}{{K^{2}_{D}} +R^{2}},$ describing transcriptional repression and the Michaelis-Menten function, $\frac {k_{4} E_{T} R}{K_{M} + R},$ describing enzymatic degradation.

The reduced stochastic model is inaccurate [27]. To investigate whether a particular step in the composite reduction leads to this inaccuracy, we compared four models: the full model, the model with reduced enzymatic degradation (ER), the model with reduced transcriptional repression (DR) and the fully reduced model obtained using both reductions (EDR) (Fig. 5a and Additional file 1: Figure S6). The solutions of these three deterministic reductions agree for a particular initial condition, which will be used for the stochastic simulation (Fig. 5b). However, when we varied the initial condition, only the ER model accurately approximated the full deterministic model (Fig. 5b). This suggests that an accurate stochastic reduction needs to contain an elementary representation of the repressor to DNA binding process. Indeed, only the stochastic ER model provided an accurate approximation (Fig. 5c-e). The deterministic QSSA pointed to the reduction step which caused the inaccuracy in the stochastic QSSA. This suggests that our theory can identify the reduction steps that lead to a valid approximation (e.g. ER model).

### Stochastic QSSA with an unbounded fast variable

Finally, we investigate an example in which the values of the fast variable are not bounded. Namely, consider the constitutive transcription of first mRNA and then translation of protein described by the deterministic system:

(21) $$  \dot {M} = \alpha_{M} - k_{M} M, ~~\dot {P} = \alpha_{P} M - k_{P} P.  $$

If *k*_*M*_≫*k*_*P*_, *M* rapidly equilibrates to its QSS (*α*_*M*_/*k*_*M*_) and the reduced model becomes

(22) $$\begin{array}{@{}rcl@{}} \dot {P} &=& \frac{\alpha_{M} \alpha_{P}}{k_{M}} - k_{P} P.  \end{array} $$

Since the system is linear, the exact variance of *P* at the steady state can be calculated [27, 55]:

$$\begin{array}{@{}rcl@{}} \sigma^{2}_{\text{full}}=\langle P\rangle\left(\frac{\alpha_{P}/k_{M}}{1+k_{P}/k_{M}}+1\right), ~~\sigma^{2}_{\text{QSSA}}=\langle P\rangle. \end{array} $$

where 〈*P*〉 is the average of *P* at the steady state (*α*_*M*_*α*_*P*_/*k*_*M*_*k*_*P*_).

**Fig. 6:**
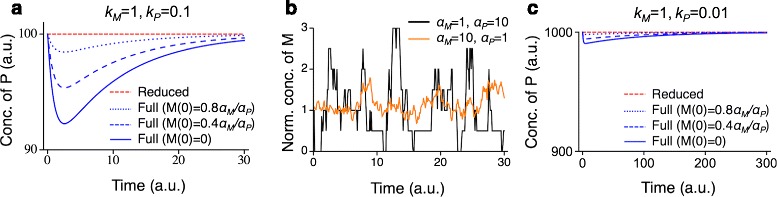
A model with an unbounded fast variable. **a** The trajectories of the full model (Eq. 21) and the reduced deterministic model (Eq. 22) during the IT period are the same for the same initial values of normalized *M*(0) by the steady state of *M* (*α*_*M*_/*k*_*M*_) when *α*_*M*_=1,*α*_*P*_=10 and when *α*_*M*_=10,*α*_*P*_=1. The difference between the trajectories increases as *M*(0) decreases. Here, *k*_*M*_=1,*k*_*P*_=0.1. **b** When *α*_*M*_=1,*α*_*P*_=10, fluctuation in the normalized *M* by its steady state is larger than for *α*_*M*_=10,*α*_*P*_=1. **c** When *k*_*P*_=0.01, the *relative* differences between the trajectories of the full and reduced deterministic model during the IT period are smaller than when *k*_*P*_=0.1 (*c.f.* panel **a**)

Previous studies note that even if *k*_*P*_/*k*_*M*_≪1, which ensures the accuracy of the deterministic QSSA, the stochastic QSSA has an error that depends on *α*_*P*_/*k*_*M*_ [27, 55]. This discrepancy can also be explained by our finding: the accuracy of the stochastic QSSA depends not only on the accuracy of the deterministic QSSA, but also on the range of fluctuations in the full stochastic system. To see how this applies to the present example, assume that *k*_*M*_ and *k*_*P*_ are fixed and *k*_*P*_/*k*_*M*_≪1. Then, as long as *α*_*M*_*α*_*P*_ is fixed, the full model (Eq. 21) always has the same reduction (Eq. 22), and the differences between trajectories of the full model and the reduced deterministic model remain the same for different initial values of normalized *M*(0) by the steady state of *M*(*α*_*M*_/*k*_*M*_) (Fig. 6a). However, a larger *α*_*P*_ (and, accordingly, a smaller *α*_*M*_) results in a lower equilibrium concentration, and hence larger fluctuation in the normalized *M* (See Fig. 6b, the coefficient of variation of *M* is $\sqrt {k_{M}/\alpha _{M}}$). This means that we need to require the deterministic reduction to be accurate for a larger range of initial conditions of the normalized *M*, and, in particular, small *M*(0)/(*α*_*M*_/*k*_*M*_) when *α*_*P*_ is large (Fig. 6a and b). Thus, while *α*_*P*_ does not affect the accuracy of deterministic QSSA, it does impact the error of the stochastic QSSA.

As *k*_*P*_ decreases, the deterministic QSSA becomes more accurate (Fig. 6c), but the difference between $\sigma ^{2}_{\text {full}}$ and $\sigma ^{2}_{\text {QSSA}}$ increases, apparently contradicting our claim. However, unlike above, the change in *k*_*P*_ leads to different reductions with different scales (Eq. 22) and therefore the accuracy of the reduction should be compared in a *relative* sense (Fig. 6a, c). Indeed, when we compare the relative accuracy of the stochastic QSSA (the coefficient of variation), we see that :

$$\begin{array}{@{}rcl@{}} \frac{\sigma_{\text{full}}-\sigma_{\text{QSSA}}}{\langle P\rangle} \approx \frac{\sqrt{\alpha_{P}/k_{M}+1}-1}{ \sqrt{\alpha_{M}\alpha_{P}/k_{P}k_{M}}} \propto \sqrt{k_{P}}, \end{array} $$

where we used *k*_*M*_≪*k*_*P*_ in the first approximation. Hence, the relative error of the stochastic QSSA decreases with decreasing *k*_*P*_, as does the relative error of the deterministic QSSA.

This example shows that the deterministic and stochastic QSSA are related as described earlier, even when the fast variable is not bounded. However, applying the simple numerical method (Eq. 8) to this example is not possible since it would require one to test the accuracy of deterministic QSSA for all possible values of the fast variables, a point we consider further in the Conclusions.

## Conclusions

Numerous methods have been developed to accelerate and simplify stochastic simulations [12–30]. The stochastic QSSA is the most widely used reduction technique due to its simplicity and general applicability [31–34, 37–47], but its validity is rarely justified rigorously [30]. Often it is tacitly assumed that the stochastic QSSA is accurate whenever its deterministic counterpart is valid [31–34]. However, recent counterexamples have brought this assumption into question [27, 30, 35, 36]. Here, we demonstrated a clear relationship between the accuracy of the two reductions. Our analysis and simulations reveal that the stochastic QSSA is valid if the deterministic QSSA is accurate over a range of initial conditions that include the most probable fluctuations (Figs. 1–6). If the deterministic QSSA is not accurate in this neighborhood, the stochastic QSSA will fail. On the other hand, if the deterministic QSSA is accurate regardless of initial conditions, the stochastic QSSA will be accurate.

We have discussed the relationship between the accuracy of the deterministic and the stochastic QSSA using common examples (e.g. Michaelis-Menten and Hill kinetics). Based on these examples, we conjecture that a similar relation holds more generally. We leave a full theoretical investigation of this conjectured relationship for future work. Thomas et al. [56] used a projector operator technique to show that, if fast reactions do not affect slow species – a condition which is not generally satisfied when the QSSA can be applied – then the propensities derived from the deterministic QSSA can be used to provide an accurate linear noise approximation. This is consistent with our result showing that the tQSSA, which holds when slow species are insulated from fast reactions, leads to a more accurate stochastic QSSA (Fig. 4). Furthermore, we considered more general cases in which the fast reactions affect the slow species, which is common in QSSA reductions (Figs. 1, 3 and 5). Our work indicates that if fast reactions do affect slow species the stochastic QSSA is more accurate when the slow species do not change much during the IT period. This indicates that the theoretical results of Thomas et al. [56] can be generalized to the case when fast reactions do affect slow species.

**Fig. 7:**
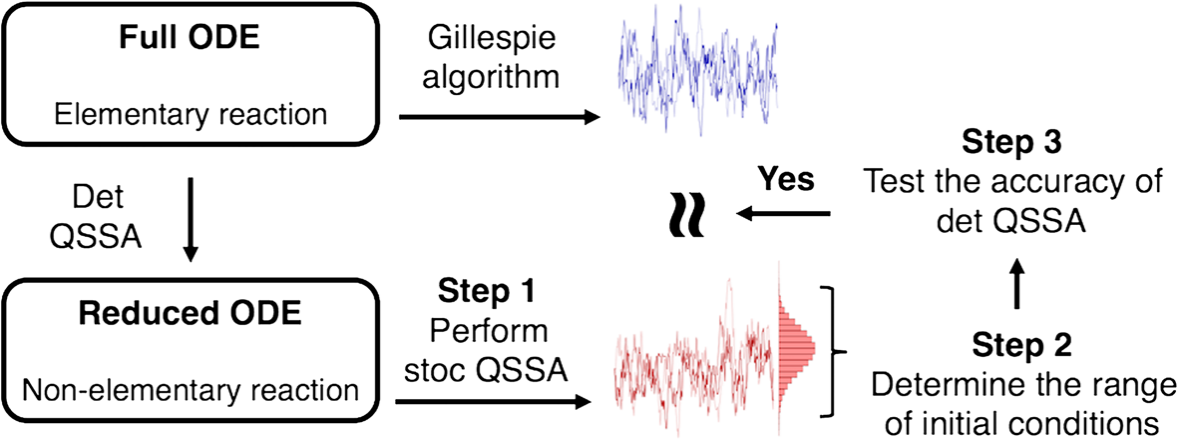
Procedure for validating the stochastic QSSA with Eq. 8. Step 1. Perform stochastic simulations with the reduced model. Step 2. From these stochastic simulations, estimate a range of initial conditions for the slow variables. For the fast variables, use all possible states for their range of initial conditions. Step 3. Using these ranges of initial conditions for the slow and fast variables, test the accuracy of deterministic QSSA. If the deterministic QSSA is accurate for all of these initial conditions, the stochastic QSSA is accurate

Based on the above relationship, we provide a simple numerical method (Eq. 8) for testing the validity of stochastic reductions that include non-elementary propensity functions (Fig. 7). In applying this method, we assume that the distribution and the range of fluctuations of the fast species is not known. Without this knowledge, we assume that fast variables can vary over all possible states, a range that may be much larger than that covered by their actual fluctuations. As a result, our method is more conservative than necessary and can produce false negatives. Our method can be improved by identifying a plausible range of initial conditions that need to be tested by estimating the actual fluctuations in the fast variables via analysis (e.g. linear noise approximation) or numerical simulation (Fig. 6b). Our approach works better if one normalizes the fast variables (Additional file 1: Figure S1), which can help reduce the parameter dependence of the fast variables’ fluctuation range.

Furthermore, to avoid stochastic simulations of the full model, we proposed to test the accuracy of the deterministic QSSA over a range of values for the slow variable determined by simulations of the reduced model (Fig. 7). It is possible that the accuracy of the deterministic QSSA inside this range does not imply validity outside the range. In such cases our method can lead to false positives and suggest the stochastic QSSA is accurate when it is not. A singular perturbation analysis of the accuracy of the deterministic QSSA can help to avoid this problem [1–3, 9, 11]. For instance, Eq. 6 shows that the accuracy of the deterministic QSSA across an arbitrary range of initial values of the slow variable (*R*(0)) is determined by the same condition. In such cases, false positives can be avoided (Fig. 2).

Diverse software packages for simulating stochastic biochemical systems are available. A common issue with all these packages is how to deal with non-elementary reaction functions. Some packages, such as STOCKS [57], require users to convert non-elementary reaction functions to elementary ones. Such simulations can be prohibitively slow. Thus other packages, such as COPASI [58], StochSS (http://www.stochss.org/) and Cain [59] allow the use of non-elementary propensity functions. The resulting simulations are faster, but may be inaccurate. Our method can provide a quick way to guarantee when such simulations can be trusted (Fig. 7). Because this method is based on our finding of a relative relationship between the error of the deterministic and stochastic QSSA (Figs. 2 and 3d-e), it could be improved if a more direct relationship can be derived.

Our work could also be extended to more general singularly perturbed systems such as reaction diffusion system [3, 60, 61]. The relation we found could also provide a bridge between theories of singularly perturbed deterministic systems [2, 3, 9, 11], and their stochastic counterparts.

## Methods

### Stochastic simulation

All stochastic simulations were performed with the Gillespie algorithm [62]. The propensity functions of both elementary and non-elementary reactions were obtained by substituting $ X=\frac {n_{X}}{\Omega }$ to the macroscopic reaction rates (Additional file 1: Table S1). *X* and *n*_*X*_ represent concentration and the number of species, respectively. *Ω* is the volume of system. We used *Ω*=1 unless it is specified. The variance and expectation values of species were estimated with 10^6^ simulations (Figs. 2 and 3d–e). The standard deviation of errors in Figs. 2 and 3d–e were estimated with the bootstrap method with 100 block length.

### Validity condition of deterministic QSSA

The deterministic QSSA of the model (Eq. 5) is accurate when a slow variable, *R*, changes little while a fast variable, *D*_*A*_, approaches its QSS in the model (Eqs. 1–3). Let us estimate how much *R* changes while *D*_*A*_ approaches its QSS. To follow the estimation method of Segel and Slemrod [1], we consider the case when *R* decays from its initial condition. The timescale of *D*_*A*_ during the IT period (*R*≈*R*(0)) can be estimated by

$$ t^{-1}_{D_{A}}=\left| \frac{\partial \dot{D}_{A}}{\partial D_{A}} \right|=k_{f} R (0) +k_{b} \  $$

Then, the relative change of *R* during the IT period becomes

(23) $$\begin{array}{@{}rcl@{}} \left| \frac{\Delta R}{R(0)} \right| &\approx& \left| \frac{\dot{R}^{\text{max}}}{R(0)} \right| t_{D_{A}} \ \\ &\approx&\left| \frac{\beta_{R} R(0)+ k_{f} R(0)D_{A}^{\text{max}}}{R(0)} \right| \frac{1}{k_{f} R (0) +k_{b}}\ \\ &=&\frac{\beta_{R}/k_{f}+ D_{A}^{\text{max}}}{R(0)+K_{D}},  \end{array} $$

where $ \dot {R}^{max}$ and $ D_{A}^{\text {max}}$ represent the maximum of $ \dot {R}$ and *D*_*A*_ during the IT period, respectively. Note that when we derived $\dot {R^{\text {max}}} \approx \beta _{R} R(0)+k_{f} R(0) D_{A}^{\text {max}}$, we assumed that mRNA evolves much more slowly than *D*_*A*_ during the IT period. Equation 23 estimates the maximal relative change of *R* during the IT period.

### Validity condition of stochastic QSSA

Our previous study showed that the stochastic QSSA of the model (Eq. 5) is accurate when: 1) binding and unbinding between *R* and *D*_*A*_ are faster than other reactions; and 2) the QSS of *D*_*A*_ of the deterministic and stochastic systems are similar [30]. When the first condition is satisfied, it was shown that replacing the fast variable *D*_*A*_ with its QSS of the stochastic system leads to an accurate reduction [16–18, 29]. Thus, when the second condition is satisfied, the stochastic QSSA becomes accurate because the stochastic QSSA uses the QSS of *D*_*A*_ driven with the deterministic system to replace the fast variables. For the first condition, we need *k*_*f*_≫*β*_*R*_=*β*_*M*_ because *K*_*D*_=*k*_*b*_/*k*_*f*_ is fixed in this study. For the second condition, the relative difference between the QSS of the deterministic (*D*_*A*_(*R*)) and stochastic ($ \left <D_{A}\right >$) systems should be small. A theorem provided in our previous study (p. 788 of [30]) shows that the difference mainly depends on the Fano factor of the fast variables (Var(*D*_*A*_)/*D*_*A*_(*R*)) and the sensitivity of the QSS solution (*dD*_*A*_(*R*)/*dR*). Since the error stems from ignoring the effect of fluctuations of the fast species on the slow species ([27, 56]), the error depends on the Fano factor of fast variables – and this error is magnified by the sensitivity of the QSS solution:

(24) $$\begin{array}{@{}rcl@{}} \left| \frac{D_{A}(R)-\left<D_{A}\right>}{D_{T}} \right| &\approx& \frac{1}{D_{T}} \frac{\text{Var}(D_{A})}{D_{A}(R)} \left| \frac{dD_{A}(R)}{dR} \right| \\\ &=& \text{Var} \left(\frac{D_{A}}{D_{T}}\right) \frac{D_{T}}{R+K_{D}} \end{array} $$

The second equality comes from Eq. 4. Under nondimensionalization, where *D*_*T*_=1, Eq. 24 becomes Eq. 7.

## Availability of supporting data

No supporting data sets are associated with this manuscript.

## Additional file

Additional file 1
**Figures S1–S6 and Table S1.** **Figure S1.** Normalization of fast variables improves the numerical method testing the accuracy of the stochastic QSSA with Eq. 8. **Figure S2.** The distribution of *R* at steady state from the stochastic simulations of the full (Eqs. 1–3) and reduced model (Eq. 5) corresponding to Fig. 2 a–b. **Figure S3.** The distribution of *S* at steady state from the stochastic simulations of the full (Eq. 9) and reduced model (Eq. 10). **Figure S4.** The accuracy of deterministic QSSAs depends on the initial conditions. **Figure S5.** Fourier transforms of trajectories obtained from stochastic simulations. **Figure S6.** Different reductions of the negative feedback loop model with enzymatic degradation. **Table S1.** The propensity functions used for the stochastic simulations. (PDF 769 kb)

## References

[1] Segel LA, Slemrod M (1989). The quasi-steady-state assumption - a case-study in perturbation. Siam Rev.

[2] Lam SH, Goussis DA (1994). The CSP method for simplifying kinetics. Int J Chem Kinet.

[3] Kaper T. Analyzing multiscale phenomena using singular perturbation methods. In: Jane C, O’Malley R, editors. Proceedings of Symposia in Applied Mathematics, vol. 56: 1999. p. 187. hardcover. ISBN-10: 0-8218-0929-6, ISBN-13: 978-0-8218-0929-7.

[4] Tzafriri R (2003). Michaelis-Menten kinetics at high enzyme concentrations. Bull Math Biol.

[5] Fall C, Marland E, Wagner J, Tyson J (2004). Computational cell biology.

[6] Ciliberto A, Capuani F, Tyson JJ (2007). Modeling networks of coupled enzymatic reactions using the total quasi-steady state approximation. PLoS Comput Biol.

[7] Bennett MR, Volfson D, Tsimring L, Hasty J (2007). Transient dynamics of genetic regulatory networks. Biophys J.

[8] Keener J, Sneyd J (1998). Mathematical physiology I: cellular physiology. Interdisciplinary applied mathematics 8/1 (2 ed.).

[9] Lee CH, Othmer HG (2010). A multi-time-scale analysis of chemical reaction networks: I. Deterministic systems. J Math Biol.

[10] Kumar A, Josić K (2011). Reduced models of networks of coupled enzymatic reactions. J Theor Biol.

[11] Goeke A, Walcher S (2014). A constructive approach to quasi-steady state reductions. J Math Chem.

[12] Kepler TB, Elston TC (2001). Stochasticity in transcriptional regulation: origins, consequences, and mathematical representations. Biophys J.

[13] Elf J, Ehrenberg MN (2003). Fast evaluation of fluctuations in biochemical networks with the linear noise approximation. Genome Res.

[14] Bundschuh R, Hayot F, Jayaprakash C (2003). Fluctuations and slow variables in genetic networks. Biophys J.

[15] Berglund N, Gentz B (2003). Geometric singular perturbation theory for stochastic differential equations. J Differ Equations.

[16] Rao CV, Arkin AP (2003). Stochastic chemical kinetics and the quasi-steady-state assumption: Application to the Gillespie algorithm. J Chem Phys.

[17] Goutsias J (2005). Quasiequilibrium approximation of fast reaction kinetics in stochastic biochemical systems. J Chem Phys.

[18] Cao Y, Gillespie DT, Petzold LR (2005). The slow-scale stochastic simulation algorithm. J Chem Phys.

[19] Haseltine EL, Rawlings JB. On the origins of approximations for stochastic chemical kinetics. J Chem Phys. 2005:123. 10.1063/1.2062048 16268689

[20] Salis H, Kaznessis YN. An equation-free probabilistic steady-state approximation: Dynamic application to the stochastic simulation of biochemical reaction networks. J Chem Phys. 2005:123. 10.1063/1.2131050 16356038

[21] Ball K, Kurtz TG, Popovic L, Rempala G (2006). Asymptotic analysis of multiscale approximations to reaction networks. Ann Appl Probab.

[22] Ullah M, Wolkenhauer O (2007). Family tree of Markov models in systems biology. IET Syst Biol.

[23] Barik D, Paul MR, Baumann WT, Cao Y, Tyson JJ (2008). Stochastic simulation of enzyme-catalyzed reactions with disparate timescales. Biophys J.

[24] Macnamara S, Bersani AM, Burrage K, Sidje RB (2008). Stochastic chemical kinetics and the total quasi-steady-state assumption: application to the stochastic simulation algorithm and chemical master equation. J Chem Phys.

[25] Crudu A, Debussche A, Radulescu O (2009). Hybrid stochastic simplifications for multiscale gene networks. BMC Syst Biol.

[26] Sanft KR, Gillespie DT, Petzold LR (2011). Legitimacy of the stochastic Michaelis-Menten approximation. IET Syst Biol.

[27] Thomas P, Straube AV, Grima R (2012). The slow-scale linear noise approximation: an accurate, reduced stochastic description of biochemical networks under timescale separation conditions. BMC Syst Biol.

[28] Crudu A, Debussche A, Muller A, Radulescu O (2012). Convergence of stochastic gene networks to hybrid piecewise deterministic processes. BMC Syst Biol.

[29] Kang HW, Kurtz TG, Popovic L (2013). Central limit theorems and diffusion approximations for multiscale Markov chain models. Ann Appl Probab.

[30] Kim J, Josić K, Bennett M (2014). The validity of quasi-steady-state approximations in discrete stochastic simulations. Biophys J.

[31] Gonze D, Halloy J, Goldbeter A (2002). Deterministic versus stochastic models for circadian rhythms. J Biol Phys.

[32] Ouattara Da, Abou-Jaoudé W, Kaufman M (2010). From structure to dynamics: frequency tuning in the p53-Mdm2 network. II Differential and stochastic approaches. J Theor Biol.

[33] Gonze D, Abou-Jaoudé W, Ouattara DA, Halloy J (2011). How molecular should your molecular model be? On the level of molecular detail required to simulate biological networks in systems and synthetic biology. Methods Enzymol.

[34] Kim JK, Jackson TL (2013). Mechanisms that enhance sustainability of p53 pulses. PLoS One.

[35] Thomas P, Straube AV, Grima R (2011). Communication: limitations of the stochastic quasi-steady-state approximation in open biochemical reaction networks. J Chem Phys.

[36] Agarwal A, Adams R, Castellani GC, Shouval HZ (2012). On the precision of quasi steady state assumptions in stochastic dynamics. J Chem Phys.

[37] Thattai M, van Oudenaarden A (2001). Intrinsic noise in gene regulatory networks. Proc Natl Acad Sci USA.

[38] Simpson ML, Cox CD, Sayler GS (2003). Frequency domain analysis of noise in autoregulated gene circuits. Proc Natl Acad Sci USA.

[39] Pedraza JM, van Oudenaarden A (2005). Noise propagation in gene networks. Science.

[40] Tian T, Burrage K (2006). Stochastic models for regulatory networks of the genetic toggle switch. Proc Natl Acad Sci USA.

[41] Scott M, Ingalls B, Kærn M (2006). Estimations of intrinsic and extrinsic noise in models of nonlinear genetic networks. Chaos.

[42] Murphy KF, Balázsi G, Collins JJ (2007). Combinatorial promoter design for engineering noisy gene expression. Proc Natl Acad Sci USA.

[43] Çağatay T, Turcotte M, Elowitz MB, Garcia-Ojalvo J, Süel GM (2009). Architecture-dependent noise discriminates functionally analogous differentiation circuits. Cell.

[44] Black AJ, McKane AJ (2010). Stochastic amplification in an epidemic model with seasonal forcing. J Theor Biol.

[45] Toni T, Tidor B (2013). Combined model of intrinsic and extrinsic variability for computational network design with application to synthetic biology. PLoS Comput Biol.

[46] Schultz D, Lu M, Stavropoulos T, Onuchic J, Ben-Jacob E (2013). Turning oscillations into opportunities: lessons from a bacterial decision gate. Sci Rep.

[47] Riba A, Bosia C, El Baroudi M, Ollino L, Caselle M (2014). A combination of transcriptional and MicroRNA regulation improves the stability of the relative concentrations of target genes. PLoS Comput Biol.

[48] Kim JK, Forger DB (2012). A mechanism for robust timekeeping via stoichiometric balance. Mol Syst Biol.

[49] Kim JK, Forger DB (2012). On the existence and uniqueness of biological clock models matching experimental data. SIAM J Appl Math.

[50] Newby J, Schwemmer M (2014). Effects of moderate noise on a limit cycle oscillator: Counterrotation and bistability. Phys Rev Lett.

[51] Glass L, Winfree A (1984). Discontinuities in phase-resetting experiments. Am J Physiol Regul Integr Comp Physiol.

[52] Locke JC, Westermark PO, Kramer A, Herzel H (2008). Global parameter search reveals design principles of the mammalian circadian clock. BMC Syst Biol.

[53] Taylor SR, Webb AB, Smith KS, Petzold LR, Doyle FJ (2010). Velocity response curves support the role of continuous entrainment in circadian clocks. J Biol Rhythms.

[54] Kim JK, Forger DB, Marconi M, Wood D, Doran A, Wager T (2013). Modeling and validating chronic pharmacological manipulation of circadian rhythm. CPT Pharmacometrics Syst Pharmacol.

[55] Shahrezaei V, Swain PS (2008). Analytical distributions for stochastic gene expression. Proc Natl Acad Sci USA.

[56] Thomas P, Grima R, Straube AV (2012). Rigorous elimination of fast stochastic variables from the linear noise approximation using projection operators. Phys Rev E.

[57] Kierzek AM (2002). STOCKS: STOChastic Kinetic Simulations of biochemical systems with Gillespie algorithm. Bioinformatics.

[58] Hoops S, Sahle S, Gauges R, Lee C, Pahle J, Simus N (2006). COPASI − a COmplex PAthway SImulator. Bioinformatics.

[59] Mauch S, Stalzer M (2011). Efficient formulations for exact stochastic simulation of chemical systems. IEEE/ACM Trans Comp Biol Bioinform.

[60] Erban R, Chapman SJ (2009). Stochastic modelling of reaction-diffusion processes: algorithms for bimolecular reactions. Phys Biol.

[61] Isaacson SA, Peskin CS (2006). Incorporating diffusion in complex geometries into stochastic chemical kinetics simulations. SIAM J Sci Comput.

[62] Gillespie DT (1977). Exact stochastic simulation of coupled chemical reactions. J Phy Chem.

