# Supplementary material for: The relationship between stochastic and deterministic quasi-steady state approximations
Source: BMC Syst Biol. 2015 Nov 23;9:87. doi: 10.1186/s12918-015-0218-3 (PMC4657384; doi:10.1186/s12918-015-0218-3)
Supplement: Additional file 1 — Figures S1–S6 and Table S1. Figure S1. Normalization of fast variables improves the numerical method testing the accuracy of the stochastic QSSA with Eq. 8. Figure S2. The distribution of R at steady state from the stochastic simulations of the full (Eqs. 1–3) and reduced model (Eq. 5) corresponding to Fig. 2 a–b. Figure S3. The distribution of S at steady state from the stochastic simulations of the full (Eq. 9) and reduced model (Eq. 10). Figure S4. The accuracy of deterministic QSSAs depends on the initial conditions. Figure S5. Fourier transforms of trajectories obtained from stochastic simulations. Figure S6. Different reductions of the negative feedback loop model with enzymatic degradation. Table S1. The propensity functions used for the stochastic simulations. (PDF 769 kb) [file 12918_2015_218_MOESM1_ESM.pdf]

Supplemental Information: The relationship between stochastic and deterministic quasi-steady state approximation

Jae Kyoung Kim,[1,2,*] Krešimir Josić,[3,4,**] and Matthew R. Bennett[5,6,***]

October 1, 2015

1: Department of Mathematical Sciences, Korea Advanced Institute of Science and Technology, Daejeon, 305-701, Korea
2: Mathematical Biosciences Institute, The Ohio State University, Columbus, OH, 43210,
3: Department of Mathematics, 4: Department of Biology and Biochemistry, University of Houston, Houston, TX, 77204, USA
5: Department of Biosciences, 6: Institute of Biosciences and Bioengineering, Rice University, Houston, TX, 77005, USA
*: Correspondence: jaekkim@kaist.ac.kr
**: Correspondence: josic@math.uh.edu
***: Correspondence: matthew.bennett@rice.edu

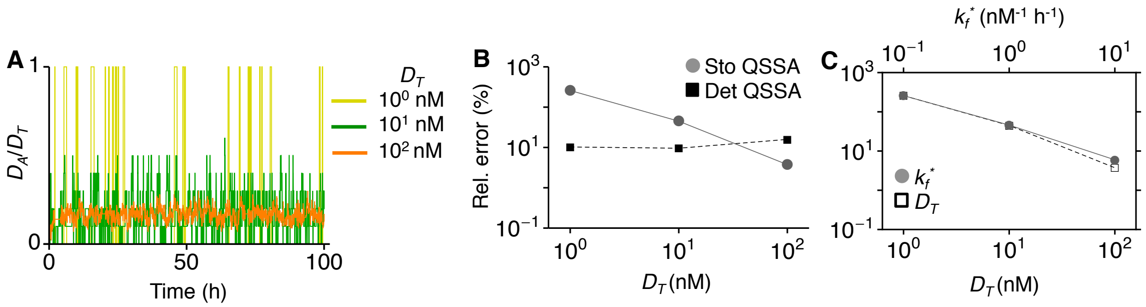

Figure S1: **Normalization of fast variables improves the numerical method testing the accuracy of the stochastic QSSA with Eq. 8**. **A**) Relative fluctuations in D_A decrease as D_T increases in the unscaled model of Eqs. 1-3. Here, the binding rate before scaling (k_f^*) is $0.1nM^{-1}h^{-1}$. Other parameters are the same as those used in Fig. 2A. **B**) Due to this change of fluctuation range, the accuracy of the stochastic and deterministic QSSA, estimated as described in Fig. 2A-B, do not change in tandem as D_T changes. Specifically; when the accuracy of the deterministic QSSA is estimated for a range of initial conditions that includes all possible values of the fast species, the error of deterministic QSSA increases as D_T increases. However, the error of the stochastic QSSA decreases. Estimating the accuracy of the deterministic QSSA for the unscaled model with Eq. 8 fails in predicting how D_T changes the accuracy of stochastic QSSA. **C**) However, this limit can be overcome with the scaled model (Eqs. 1-3). In Fig. 2A, as k_f increases, the stochastic QSSA of the scaled model (Eqs. 1-3) becomes more accurate. Since $k_f = D_T k_f^*$, the result of Fig. 2A indicates that either increasing D_T or k_f^* reduces the error of the stochastic QSSA for the unscaled model, matching simulations of the unscaled model. This shows that the results of the scaled model can be converted to those of the unscaled model.

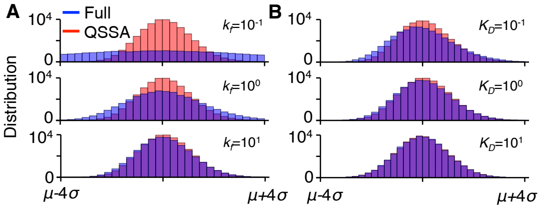

Figure S2: **The distribution of R at steady state from the stochastic simulations of the full (Eqs. 1-3) and reduced model (Eqs. 5) corresponding to Figs. 2A-B**. As k_f (A) and K_D (B) increase, the difference between the distributions of the full and reduced models decrease just as do the coefficient of variation (*c.f.* Figs. 2A-B). Here μ is the mean of R and σ is the standard deviation of R

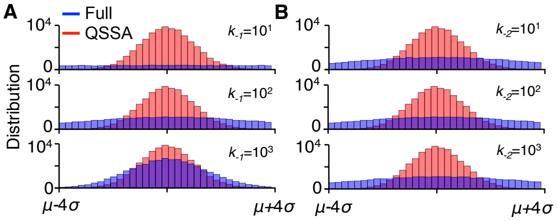

Figure S3: **The distribution of S at steady state from the stochastic simulations of the full (Eq. 9) and reduced model (Eq. 10)**. **A**) When k_{-1} increases the difference between the distributions of the stochastic QSSA and the full model decreases, just as does the coefficient of variation (Fig. 3D). **B**) When k_{-2} increases the differences of distributions of stochastic QSSA and full model show little change, similar to the behavior of the corresponding coefficient of variations (Fig. 3E). Here μ is the mean of variable S and σ is the standard deviation of S.

I

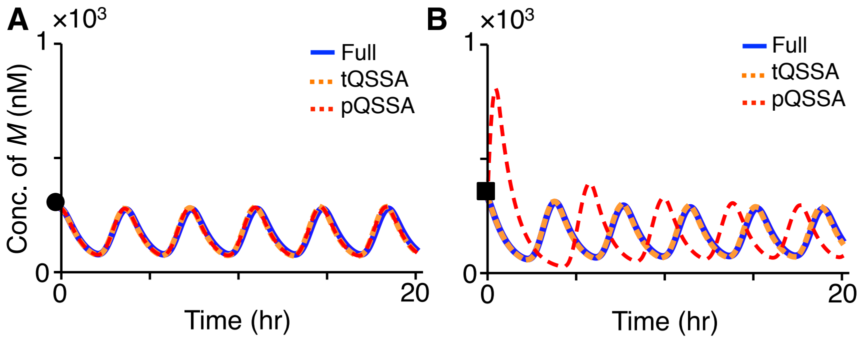

Figure S4: **The accuracy of deterministic QSSAs depends on the initial conditions. A)** When the initial condition is on the limit cycle (the black circle in Fig. 4C), both the deterministic pQSSA and tQSSA accurately approximate the full model of transcriptional negative feedback (Fig. 4A). **B)** On the other hand, for initial conditions off the limit cycle (the black square in Fig. 4C), only the deterministic tQSSA accurately approximates the full model.

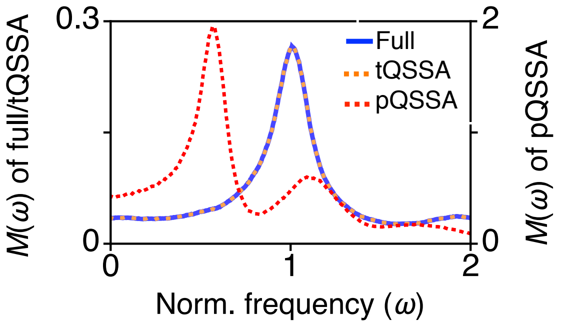

Figure S5: **Fourier transforms of trajectories obtained from stochastic simulations**. Fourier transforms of trajectories of the stochastic model (10^4 cycles) obtained using the tQSSA (Eq. 17) match those of the full model (Eqs. 11-14). However, Fourier transforms obtained using the pQSSA (Eq. 18) show oscillations with a lower frequency and high amplitude than those from the full model, as seen Fig. 4B.

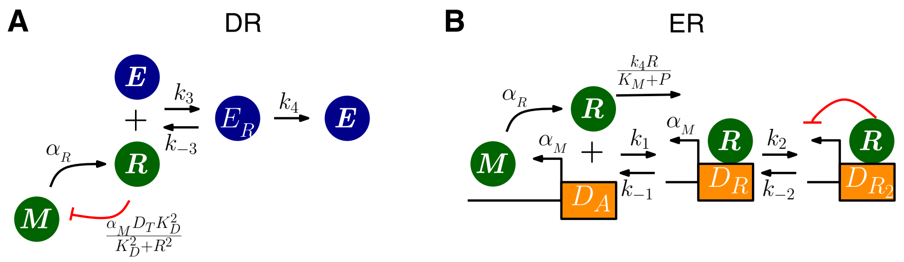

Figure S6: **Different reductions of the negative feedback loop model with enzymatic degradation A)** The diagram of the DR model where transcriptional repression step only is reduced. **B)**The diagram of the ER model where enzymatic degradation step only is reduced. See Fig. 5A for the diagram EDR model where both the transcriptional repression step and enzymatic degradation step are reduced.

Table S1: The propensity functions used for the stochastic simulations

Reaction	Macroscopic rate	Propensity function
$\xrightarrow{k} X$	k	k
$X \xrightarrow{k} Y$	kX	$k\frac{n_X}{\Omega}$
$X + Y \xrightarrow{k} Z$	kXY	$k\frac{n_X}{\Omega}\frac{n_Y}{\Omega}$
$X \xrightarrow{kZ} Y$	$\frac{kX}{K_M+X}$	$\frac{kn_X/\Omega}{K_M+n_X/\Omega}$
$X \xrightarrow{kZ} Y$	$\frac{kX^2}{K_M^2+X^2}$	$\frac{k(n_X/\Omega)^2}{K_M^2+(n_X/\Omega)^2}$
